# Supplementary material for: Are carbon emissions trading and green financial instruments synergistic? -Comprehensive quantitative research based on content analysis
Source: PLoS One. 2024 Mar 7;19(3):e0298601. doi: 10.1371/journal.pone.0298601 (PMC10919601; doi:10.1371/journal.pone.0298601)
Supplement: S1 Data — (ZIP) [file pone.0298601.s001.zip › Data/地级市Z_F工作报告.zip!/┴╔─■/╕╖╨┬╩╨/└┤╫╘╣1⁄2╓┌║┼í╛┬φ┐╦╩2╛▌═°í┐_2004.docx]

各位代表：

　　现在，我代表市人民政府，向大会作政府工作报告，请予审议，并请市政协各位委员和其他列席同志提出意见。

　　一、2004年政府工作回顾

　　2004年，全市各族人民在中共阜新市委的领导下，以邓小平理论和“三个代表”重要思想为指导，紧紧围绕经济转型这条主线，团结拼搏，开拓奋进，较好地完成了市十三届人大二次会议确定的主要工作任务。这一年，是阜新经济发展史上重要而不平凡的一年，是我市经济连续实现跨越式发展的一年，是经济转型试点工作取得重大阶段性成果的一年。

　　——国民经济保持强劲发展势头。预计全市生产总值完成127.1亿元，增长21%，其中，一、二、三产业分别增长30.8%、19.4%和16.2%；财政一般预算收入6.25亿元，增长22.3%。

　　——固定资产投资和消费有较大幅度增长。预计全社会固定资产投资完成58亿元，增长21.1%；社会消费品零售总额实现57亿元，增长14.2%。

　　——开放型经济实现新突破。直接利用外资2586万美元，增长16%；出口创汇3654万美元，增长50.2%。

　　——城乡居民收入增长较快。预计城镇居民人均可支配收入5650元，增长9.9%；农民人均纯收入2850元，增长41.9%。

　　过去的一年，市政府工作主要体现在以下十个方面。

　　1、转型振兴项目攻坚战取得明显成效，经济转型向纵深推进。去年，是我市项目建设力度最大的一年。全市实施投资1000万元以上重点项目168个，总投资179亿元。阜新发电厂三期技改、阜新金山54万千瓦煤矸石热电、阜矿集团热电厂三期扩建、金隆木业木材加工和鹰山水泥一期改造等21个投资1亿元以上的大项目开工建设，双汇百万头生猪屠宰线、沈阳乳业彰武分公司2万头奶牛基地一期工程等31个投资3000万元以上的项目竣工。23个经济转型重点项目中，市原种猪场二期工程等3个项目竣工，退耕还林工程、农网建设与改造二期工程等19个项目正在建设，引白工程正在做开工前准备。加快培育和发展以农产品深加工为重点的接续主导产业，阜新双汇形成6.5万吨低温火腿肠生产能力，阜新大江形成年屠宰1200万只肉鸡能力，阜新伊利乳业形成日处理鲜奶100吨能力，一批龙头企业规模不断扩大；山东东阿阿胶集团等龙头企业落户阜新。阜新被中国食品协会确定为全国13个食品工业强市(县)之一。

　　2、农业结构调整取得突破性进展，农业生产创历史最好水平。粮食总产量158万吨，增长43.4%。特色种植业面积占总播种面积34%。畜牧业产值达到农业总产值的45%。为农业产业化龙头企业配套的基地建设取得新突破。全市新增瘦肉型商品猪32.5万头，奶牛饲养突破2万头大关，新增肉鸡生产能力1000万只。五大示范工程继续推进，落实无公害农产品基地208万亩，新建“四位一体”生态模式户5761户，新发展蔬菜保护地7000亩，林下经济开发50万亩。植树造林75.9万亩，其中，退耕还林65万亩。对退耕还林地补植46.8万亩。封山禁牧工作全面启动。完成以东北黑土区综合防治试点为主的水土流失治理面积35.6万亩，新增有效灌溉面积5.9万亩。机械化保护性耕作面积56.5万亩。人工增雨8亿立方米。

　　3、工业结构进一步优化，工业经济实现持续高速发展。规模以上工业完成产值100.9亿元，增长22.3%；实现利润1.2亿元，为上年同期的1.3倍。阜矿集团井工矿原煤产量历史上首次突破1000万吨。全市实施技改项目66项，技改投资10亿元，增长29.9%。恒瑞公司500吨浮法玻璃生产线等15个项目达产达效，高强力运输带、仙鹤制药等11个项目竣工投产。玻璃、橡胶、氟化工、电子工业园区建设取得新进展。阜新东新电容器双向拉伸聚酯薄膜等3个项目列入国家老工业基地振兴项目。25户国有企业完成转制，盘活资产7.8亿元。阜新市鹰山水泥厂和氟化学公司分别被大连永盛公司和锦州天合公司成功收购，香港力劲科技集团收购北方压铸机产权，阜新电缆厂与河北宝丰集团实现合资合作。7户企业列入国家破产计划。王营矿、矿山机械厂破产进展顺利。

　　4、招商引资势头良好，对外开放再创新水平。以项目为核心，突出重点国家和地区，充分利用六个载体、十四个渠道进行招商引资。全市招商引资到位31亿元，增长19.2%。共引进外资项目43个，合同额1.1亿美元，是上年的3.7倍。组织了赴日本、厦门、沈阳、武汉、上海、江浙等6次大规模招商活动。台湾幸松公司投资的涤纶纺织项目、香港富年公司投资的房地产开发项目等一批利用外资项目开工建设。积极调整出口产品结构，出口创汇创历史最好水平。阜新经济技术开发区招商引资力度进一步加大，新入驻企业65户。

　　5、县区经济迅猛发展，民营经济实现新的突破。县区生产总值占全市的比重由上年的44.9%提高到50%。两县五区、阜新经济技术开发区和阜新国家农业科技园区生产总值的增幅均超过全市平均水平，平均增幅高于全市9.7个百分点。全市民营经济实现营业收入157亿元、实缴税金3.6亿元、从业人员23.6万人，分别增长了29.9%、30.4%和13.6%。新增销售收入超千万元的民营企业49户，其中，超5000万元的14户，超亿元的4户。全市乡镇企业增加值、实缴税金分别增长42.8%和39.3%。

　　6、市场体系建设步伐加快，第三产业稳步发展。大商集团阜新新玛特购物广场、新玛特西山超市竣工开业，阜新商贸城投入使用，阜新建筑装饰材料市场建成。完成中小型商业企业改革预案。粮食购销市场化初步形成。供销社系统新建“新合作”连锁超市20家。矿区特色旅游进入规划论证阶段，海棠山、瑞应寺旅游开发取得新的进展，旅游总收入和外汇收入分别增长21%和38%。财政、审计部门在加强资金调度、强化经济转型项目资金监管等方面作出了积极贡献。全市金融机构为地方企业核销贷款本息6亿元，并对经济转型重点项目继续给予了大力支持。

　　7、城市建设“十大工程”全面推进，城乡人居环境明显改善。完成46条小街小巷改造，为230栋旧楼铺设了连街甬道，对29条街路进行路灯改造，主要街路基本消灭无灯路。完成供水管网改造16公里，解决3084户群众吃水难问题。新增煤气用户5276户，新增供热面积75万平方米。对城市南部平房区改造面积达180万平方米。棚户区改造工程启动，完成拆迁面积20.7万平方米。累计归集住房公积金4.5亿元，8.7万名职工实现个人帐户管理。垃圾处理厂、新长途客运站投入使用，海新路七号桥主体工程完工，污水处理厂开工建设。细河治理工程通过省验收。城市植树32.3万株，新增城市绿地面积101万平方米。站前区域综合整治取得初步成效。对市区26条街路实行规范化管理。采煤沉陷区治理工程79栋楼房竣工并交付使用，4375户沉陷区居民喜迁新居。海州矿排土场复垦工程正式开工。完成村通油路412公里，72个行政村实现通油路。小城镇建设取得新的进展。

　　8、就业再就业工作取得新成效，社保体系进一步完善。坚持就业优先，实现就业和再就业4.7万人。劳务输出15.8万人，其中，输出城市下岗职工1万人。市人力资源大厦开工建设。基本养老保险费征缴1.9亿元，增长15%，离退休人员养老金社会化发放率和个人帐户基金做实率均达到100%；失业保险参保人数22.9万人，增加3万人；医疗保险人数27万人，增加3.8万人。全市低保补贴标准人均提高10元。全市发放救助金1300万元，救助城乡贫困户7.1万户。为农村特困户建房150套。全市95.6%的社区“一站三室”达标，社区干部工资补贴有了新提高。

　　9、科技教育健康发展，社会各项事业全面进步。继续加强与高等院校、科研院所的科技合作，引进推广农业新品种112个、新技术18项。科技部“阜新现代农业科技专项”已带动资金投入1.9亿元。实现高新技术产值12.5亿元，增长22.5%。完成专利申请237件，增长26%。IT产业迅速发展，330户企业总营业收入突破亿元。阜新国家农业科技园区被批准为国家农产品加工示范区及国家级农业标准化示范区。合理调整中等职业教育布局，组建了市第三职业中专。调整农村中小学91所，新建九年寄宿制学校5所，85%的农村初中达到省级标准化。对4.7万名农村贫困户和城市低保户家庭学生实行免费义务教育。改造危险校舍13.7万平方米。实施聘请国外专家项目18项，聘请国外专家20人。基本实现了全市一村一名大学生的预期目标。市县两级疾病预防控制中心建成使用，紧急救援中心综合楼落成。专业艺术文化取得新辉煌，现代京剧《血胆玛瑙》在省第六届艺术节上荣获11项金奖。群众性文化体育活动蓬勃开展。篮球城建设取得新成果，成功承办了全国青年男篮联赛。农村有线电视村村通入户8万户，数字电视工程正式启动。依法规范新闻出版市场，收缴非法出版物2万册（盘）。省农村部分计划生育家庭奖励扶助制度在我市的试点工作取得成效。妇女、儿童、老龄、残疾人事业和物价、档案、地方志、防震减灾等工作取得新进展。

　　10、民主法制建设取得新进展，政府自身建设不断加强。自觉接受人大的法律监督和政协的民主监督，及时办理人大代表议案、建议和政协提案，办结率100%，满意率90%以上。完成了第八届村委会换届选举工作。实现“四五”普法实施阶段的工作目标。社会治安秩序进一步好转，全市刑事案件发案率比上年下降6.3%。在全市集中开展了解决突出信访问题、化解社会矛盾纠纷专项治理工作。维护市场经济秩序，全市清理无照经营6800户。深入开展“环境建设年”活动，全市行政审批事项由1100多项减少到210项。市行政服务中心正式成立，两批39个部门、168项审批项目及44项服务项目进入中心。狠抓执法队伍建设，清理不合格行政执法人员1521人。认真落实廉政建设责任制，加大了对违法违纪案件的查处力度。经济普查工作全面展开。军民共建取得新成效。民族宗教、外事侨务、对台事务、国家安全、国防教育、人防民防等工作进一步加强。

　　国税、地税、工商、质量技术监督、药品监督、出入境检验检疫等中省直部门在地方经济发展中发挥了积极作用。阜矿集团、阜新发电厂、阜新电业、烟草、邮政、网通、移动、联通、保险、石油公司等中省直单位为地方经济发展作出了突出贡献。

　　各位代表，过去的一年，阜新经济转型工作得到了党中央、国务院和省委、省政府的充分肯定。温家宝总理在振兴东北等老工业基地工作座谈会上明确指出，资源型城市转型试点工作有序推进，而且看到了效果。要求阜新进一步抓好转型试点，加快发展步伐，为振兴东北老工业基地提供积极的示范。省委书记李克强在阜新调研时强调，阜新这样一个以煤炭为主要依托的城市，已经探索到了一条振兴的道路，希望看到阜新转型更大的成果。我们要不负厚望，不负重托，努力在阜新转型复兴的伟大征程中迈出更加坚实的步伐！

　　过去一年我们取得的成绩，是在市委的正确领导下，在市人大、市政协的监督支持下，全市人民团结奋斗，顽强拼搏的结果。在此，我代表市人民政府，向全市各族人民致以崇高的敬意！向给予我们大力支持的人大代表、政协委员、各民主党派、工商联、无党派人士、人民团体、中省直单位、驻阜部队、武警官兵及社会各界人士表示衷心的感谢！

　　回顾一年来的工作，我们既要看到已经取得的成绩，又要清醒地认识到前进道路上还存在着一些不容忽视的矛盾和问题。长期积累的结构性、体制性、机制性矛盾还没有从根本上得到解决，转型发展的任务十分繁重；融资渠道少，投入总量不足，对经济转型具有牵动力的大项目不多；产业、产品结构亟待升级，国有企业改革任务仍然很重；财政依然十分困难，收支矛盾愈加突出；就业和社会保障的压力依然较大，城乡部分群众生活还很困难；个别政府工作人员存在办事不公、以权谋私、损害群众利益的现象，工作作风还需要进一步改进。对于这些问题，我们已经高度重视，在今后工作中，要进一步采取有效措施，认真加以解决。

　　二、 2005年政府工作安排

　　今年，是“十五”计划的最后一年，也是我们抓住新一轮发展机遇，将阜新转型复兴的良好局面向深度和广度推进，实现新一轮超常规、跨越式发展的关键一年。国家建立资源开发补偿机制和衰退产业援助机制，决定在阜新先行试点，为阜新经济转型带来了又一次难得的历史性发展机遇。如果把转型试点初期的23个重点项目比喻为经济转型的第一级火箭，那么，“两个机制”在阜新先行试点将是推进阜新经济转型的第二级火箭。第二级火箭推力更大，带动更加长远。省委、省政府继续对阜新经济转型给予高度重视和特殊支持，近期将作出《关于加快推进阜新经济转型的决定》。阜新经济转型三年的探索和实践，进一步坚定了阜新人民加快推进经济转型的信心和决心。全市上下对经济转型的认识不断深化、思路更加清晰，形成了万众一心、团结奋进的局面。我们要牢牢把握新一轮发展机遇，进一步增强推进经济转型试点工作的责任感和使命感，励精图治，乘势前进，把阜新经济转型推向一个新的阶段。

　　2005年政府工作的总体要求是：以邓小平理论和“三个代表”重要思想为指导，认真贯彻党的十六大和十六届三中、四中全会精神，全面落实市委九届八次全会部署，牢固树立科学发展观，紧紧围绕经济转型这个中心，突出结构优化、扩大开放、深化改革三个重点，实施项目牵动战略，大力发展农产品加工业、比较优势产业、县区经济和民营经济，加快构筑“主导产业+支柱产业”的经济格局；坚持以人为本，扩大就业再就业，完善社会保障体系，着力解决人民群众生产生活中的实际问题；加强社会主义政治文明和精神文明建设，加快发展各项社会事业，努力构建和谐阜新，推进阜新经济实现新一轮超常规、跨越式发展。

　　2005年国民经济和社会发展主要预期目标是：

　　——保持经济快速增长。全市生产总值增长18%，奋斗目标为增长20%以上；地方财政一般预算收入增长15%，奋斗目标为增长20%以上。

　　——增强经济发展后劲。全社会固定资产投资增长22%以上。

　　——提高对外开放水平。直接利用外资增长35%以上；出口创汇增长20%以上。

　　——创造更多的就业岗位。新增城镇实名制就业4.1万人；城镇登记失业率控制在6%以内。

　　——努力增加居民收入。城镇居民人均可支配收入增长10%以上；农民人均纯收入增长20%以上。

　　为实现上述目标，今年政府工作要把握好以下几个关键问题：

　　第一，必须始终坚持以经济转型为主线。经济转型是阜新实现伟大复兴的唯一选择。要始终把经济转型放在统领全局的位置，一切围绕转型，一切突出转型，一切服务转型。要抓住新一轮发展机遇，积极争取“两个机制”试点早日在阜新实施，推进阜新经济转型向纵深发展。

　　第二，必须坚持全面、协调、可持续的科学发展观。紧密结合阜新的实际，统筹经济发展。在加快经济发展的同时，提高经济运行质量，重视环境保护，解决好阜新转型发展中的各种深层次矛盾，解决好经济社会协调发展的薄弱环节，使阜新的发展步入良性循环轨道。

　　第三，必须大力推进经济结构的优化。立足现有基础，发挥资源优势和产业比较优势，大力实施项目牵动战略。通过引进和新上一批对经济转型有牵动力的大项目，形成产业集群优势，促进经济结构优化和综合竞争力的提升。

　　第四，必须坚持以改革开放为强大动力。以深化改革来着力解决长期积累的体制性、机制性矛盾。充分利用国际国内两个市场、两种资源，进一步扩大对内对外开放。要在改革中解决发展中的问题，在开放中谋求更快的发展。

　　第五，必须坚持不懈地优化经济发展软环境。从完善制度和强化监督入手，狠抓软环境薄弱环节，大力营造以企业为重点的发展环境和以项目为重点的投资环境，以一流的经济发展软环境，增创一流的经济发展新优势。

　　第六，必须坚持以惠及全市193万人民为宗旨。坚持执政为民，把工作的着力点放到研究解决群众生产生活中的紧迫问题上，倾听群众呼声，关心群众疾苦，切实保障城乡困难群众的基本生活，让广大人民群众共享经济转型的成果。

　　全年要重点抓好以下十个方面工作。

　　（一）实施项目牵动战略，加快推进经济转型向纵深发展

　　项目是阜新的生命线，是转型之基，振兴之本。要抓住当前的有利时机，充分利用国家实施东北老工业基地振兴战略和经济转型试点的政策优势，以“项目建设年”为载体，通过实施项目牵动战略，实现我市项目建设的新跨越。要突出重点，全力抓好投资3000万元以上的210个大项目。其中，招商引资签订90个，开工建设76个，竣工投产44个。通过加快项目建设，优化工业经济结构，全地区工业总产值增长28%，其中，规模以上工业总产值增长23%。

　　按照培育接续主导产业的要求，壮大一批农产品加工龙头企业项目。扩大肉制品加工规模和生猪屠宰能力，阜新双汇年销售收入力争达到15亿元；阜新大江实现年加工肉鸡1200万只；阜新伊利乳业实现日处理鲜奶200吨；美中鹅业形成年屠宰白鹅600万只能力；振隆公司、北方毛革、大洋木业、金隆木业、香香食品、天诚食品、田园实业等龙头企业扩大生产能力；阜新东阿阿胶加工和阜新草原兴发绿鸟鸡、羔羊生产线项目开工建设。继续引进一批产业关联度大、技术装备水平高、带动能力强的龙头企业落户阜新。

　　按照发展壮大支柱产业的要求，抓好一批新建续建项目。继续抓好稳煤强电。抓紧推进五龙矿井改扩建项目建设，力争上半年开工建设综合开发内蒙古白音华煤田项目；加快推进阜新发电厂三期改造、阜新金山54万千瓦煤矸石热电和阜矿集团热电厂三期扩建工程建设，开工建设双益热电和群明热电项目，争取彰武火电项目立项、金山风电二期开工。大力培植骨干企业，集中力量抓好一批投资超千万元的工业新建续建项目。开工建设鹰山水泥日产2500吨新型干法水泥熟料项目和生产线二期改造等一批新建项目；重点抓好超懿集团年产350万米精纺面料、石油机械公司年产1.6万台（套）潜油螺杆泵等一批续建项目；确保祥合水泥二期、大金集团三期等一批重点技改项目的投产达效。同时，依托轻纺工业的优势，新上一批服装、纺织等劳动密集型项目。加大矿产资源勘探力度，推进一批矿产资源开发利用项目。

　　以工业园区为载体，推进一批项目建设。抓好恒瑞科技公司的建筑平弯钢化玻璃、玻璃工艺品等玻璃深加工项目建设，做好1000万平方米超薄电子玻璃基板项目的国债申报工作；抓好双向拉伸聚酯薄膜生产线、中小功率电力半导体器件及宽带网用通信电缆项目；金凯化工年产550吨氟化物项目竣工投产，开工建设年产200吨氟碳醇及1000吨超微细粉末聚四氟乙烯树脂项目；抓好300万平方米输送带二期工程；推进机械加工园区建设。

　　（二）以农民增收为出发点和落脚点，大力推进农业结构战略性调整

　　农业和农村工作要按照统筹城乡发展的思路，围绕农民增收，突出重点、突出特色、突出项目，重点发展畜牧业、特色种植业、林果业和设施农业，整体推进农业结构战略性调整。

　　积极推进农业结构调整，进一步提高农业产业化水平。实行严格的耕地保护制度，切实提高粮食综合生产能力，不断增强农业抵御自然灾害能力。要把发展畜牧业作为农业结构调整的重中之重，按照分类推进的原则，加大瘦肉型猪、奶牛、肉鸡等14个农产品基地建设，壮大基地规模，形成带动效应。新发展饲养大户1000个，新增瘦肉型猪30万头；新增奶牛基地20个，奶牛饲养量达到3万头；肉鸡饲养量达到1200万只；白鹅饲养量达到850万只；肉兔饲养量达到40万只。新增牧草基地面积7.5万亩。新植果树2万亩。加快发展设施农业，大力发展无公害无污染绿色食品生产和以油料、药材、瓜菜、小杂粮为主的区域特色种植业，经济作物面积达到140万亩。

　　以户均一个致富项目为载体，全面启动百万农民增收致富工程。以整乡、整村、整组整体开发为主，实行集中与分散相结合，全方位挖掘和拓宽农民增收渠道。年内从事致富工程项目的户数要达到10万户。积极引导和培育农村经济合作组织。要把劳务输出作为农民增收的主渠道，强化各级劳务输出工作体系建设，全年农村劳务输出达到15万人次以上。

　　继续推进五大示范工程和四大体系建设。农业生产标准化在去年示范的基础上推广1万亩；以杂粮和薯类作物为重点的有机食品示范工程面积260亩；林下经济示范点50个，开发面积60万亩；

　　“四位一体”生态模式户示范和节水示范要扩大范围和规模。继续完善良种繁育、绿色食品检测、技术服务推广和疫病防治四大体系建设。“四线百园”建设要有新的进展。

　　加快推进生态环境和农业基础设施建设。人工造林73.7万亩，其中，退耕还林60万亩，“三北”造林10万亩。全面落实封山禁牧各项措施。以东北黑土区水土流失综合防治试点为重点，完成水土流失治理面积25万亩，建设389处拦蓄地表水工程，完成防氟改水工程171处。全力完成机械化保护性耕作任务，推广新机具500台。

　　（三）提升改造传统服务业，加快旅游业发展步伐，构筑具有阜新特色的现代服务业

　　把发展第三产业作为扩大就业、优化结构、推进转型的重要举措，全社会消费品零售额要达到66亿元，增长15%以上。进一步完善市场体系建设，信息产业大厦主体完工，建筑装饰材料市场启用，新建和改建蔬菜批发市场、果品批发市场、电子商城等一批大型专业市场，引进麦当劳等国内外知名品牌，带动全市商贸、餐饮等传统服务业的提升改造。依托资源优势，启动矿区特色旅游，加快瑞应寺修复，完善海棠山、大清沟、查海遗址等景区建设。规范发展会计、律师、评估、咨询、信息等新兴服务业，逐步形成活跃有序的中介服务市场。结合棚户区改造，加大房地产业开发力度，年内开发170万平方米，竣工80万平方米。

　　充分发挥财税金融对经济转型的资金支撑作用。要千方百计抓好财源建设，积极培育新的税收增长点。坚持依法治税，加大征管力度，加强非税收入管理，确保财政收入稳定增长。对政府各种财政性资金、国有资产类资源等财力资源进一步整合，提高政府可支配财力规模，最大限度地提高资金使用效益。广开融资渠道，积极争取上级资金支持经济转型重点项目。

　　要切实加强金融监管，改进金融服务。继续抓好银行不良贷款核销工作；继续搞好银企对接，加大对重点项目的信贷支持力度，增加有效投入，为地方经济建设提供强有力的金融支持。

　　（四）加大扶持力度，构筑发展平台，实现县区经济和民营经济新的跨越

　　县区经济是我市经济新一轮超常规、跨越式发展的重要增长极。要全面落实省、市关于支持县域经济发展的政策措施，以工业化为核心，以开放为突破口，以农民增收、工业增效、财政增长、后劲增强为目标，主攻民营经济，培育特色经济，提升劳务经济，发展小城镇经济，构建县区经济发展的平台，做大做强县区经济总量。要把发展民营经济作为壮大县区经济的战略举措，作为各级政府的重要工作，进一步确立民营经济与其他经济成份平等竞争的市场主体地位，鼓励民营企业参与国有企业改革和实施股份制改造，实现国企民企双进、双强、双赢。要进一步营造环境，加大政策扶持、金融服务、依法保护和环境治理力度，集中扶持一批民营骨干企业，培育壮大一批民营优势产业集群，鼓励发展一批中小民营企业。全年销售收入超5000万元的民营企业达到20户，超亿元的10户。乡镇企业增加值、实缴税金、从业人员均增长20%以上。

　　（五）以招商引资为重点，全方位扩大对内对外开放

　　把招商引资作为对外开放的重中之重，全年招商引资要实际到位37.2亿元，增长20%以上。继续抓好六个招商载体建设，不断完善和利用好十四个招商引资渠道，选择一批农业产业化、工业结构调整、基础设施、城区改造、现代服务业等项目，进行高质量项目包装，积极向外推介，促成一批招商项目。要紧紧盯住上海、温州、东莞等发达地区和山东、河北、河南等产业聚集地区，抓住日、韩产业调整机遇，引进和承接一批产业转移、产业辐射项目。要充分发挥资源优势，引进一批用电量大的项目和食品加工业项目。要主动加入沈阳经济区，利用好沈阳招商平台，实行错位发展，承接和引进一批项目。要特别抓好协议项目和合同项目的跟踪落实，提高资金到位率和开工投产率。强化对现有外资企业的服务，促进企业增资扩股和以商招商。大力调整出口产品结构，积极推进出口市场多元化，努力提高出口产品竞争力。积极开拓国际劳务市场，继续开展对外承包工程和劳务合作。阜新经济技术开发区要扩大引资规模，提升引资质量和水平，力争在上大项目上取得新的突破。

　　（六）进一步深化改革，加快推进体制、机制创新

　　围绕推进转型发展，继续深化各项改革。要以产权制度改革为突破口，加快中小国有企业股份制改造，实现投资主体多元化。年内统计口径内一般竞争性国有工业企业基本完成转制。认真组织好海州矿、高德矿、矿山机械厂、冶金备件厂等企业的关闭破产和重组工作。积极做好企业上市融资工作。要巩固扩大农村税费改革成果，坚决防止农民负担反弹。推进土地承包经营权的流转，发展适度规模经营。完善“四荒”使用权拍卖管理办法，完成林业、水利产权制度改革。进一步深化供销社改革，大力发展农村中介组织。农村信用社改革完成阶段性目标。鼓励我市商贸企业加盟国内外大型流通集团。建立健全粮食直补制度，放开和规范粮食市场。年内30%流通企业实现转制。按照“统筹规划、突出重点、完善政策、分类指导、稳步推进”的原则，做好事业单位改革工作。

　　（七）大力推进城乡基础设施建设，进一步完善城市整体功能

　　继续加强城乡道路交通网络建设，逐步提升区位优势。阿金公铁立交桥年内主体完工，彰武至沈阳、阜新至四平和阜新至朝阳三条高速公路正式开工，新乌铁路尽快立项批复、开工建设，完成创业路、东风路、海新路、高孙路等主干路改造，建设县级以上干线公路75公里。重点推进城市地下管网改造，改造供水管网44.6公里、供热管网15.1公里，改造燃气10.5公里中低压管网和39.7公里燃气引入管。争取年内开工建设引白水源工程。加大矸石山复垦和搬迁后的采煤沉陷区土地治理、复垦力度，启动海州露天煤矿闭坑后综合治理工程。在继续搞好土地使用权拍卖的基础上，进一步拓宽经营城市的领域，重点对道路、桥梁、广场、灯箱等公用设施的冠名权、广告设置等城市无形资产向社会公开招标拍卖。按照统筹城乡发展的要求，加快重点镇、示范镇的建设。

　　（八）实施“五大民心工程”，认真解决群众生产生活中的热点难点问题

　　要通过营造稳定的社会环境、良好的治安环境和公正的法治环境，努力建设和谐阜新。要把维护好、实现好、发展好人民群众的根本利益作为一切工作的出发点和落脚点，通过实施“五大民心工程”，把老百姓关心的热点难点问题放到更加突出的位置上来。

　　实施就业再就业工程。继续坚持就业优先，努力改善就业环境，落实好就业再就业各项优惠政策。把扩大就业和再就业有机地体现在经济转型、结构调整、深化改革、扩大开放等各项工作中去。以解决并轨失业人员、“4050”人员和沉陷区搬迁的下岗失业人员再就业为重点，采取灵活多样的方式，努力拓展就业的空间和领域。进一步完善就业服务体系建设，年内培训下岗失业人员2万人次。市人力资源大厦投入使用。

　　实施社会保障工程。加快基本养老保险市级统筹步伐。加强失业保险基金的管理和失业保险参保扩面工作。抓好医疗保险扩面，医保基金征缴率要达90%以上。加强对低保对象的动态管理，加快建立城市低保户中的特困户重大疾病医疗救助制度。建立农村低保制度，将4.5万农村特困人口纳入保障范围。强化住房公积金管理，满足中低收入家庭购房需求。以开展“服务明星”活动、社区文化节活动为载体，深入开展“社区服务年”活动。加强社区卫生医疗机构建设，方便社区居民就医。

　　实施扶贫帮困工程。动员全社会力量搞好扶贫开发，解决城乡特困人口基本生活问题。做好实施整体推进扶贫的118个项目村的巩固提高工作，完成移民扶贫360户。继续搞好农村“五保户”等困难群体救助工作。广泛动员社会力量捐资助学，资助义务教育阶段城市困难低保户和农村贫困户家庭子女就学7万人次。改善农村中小学办学条件，改建农村中小学危险校舍3万平方米。

　　实施改善居民生活环境工程。继续抓好小街小巷治理，全部完成城市中心区旧楼前铺设甬道工程，城市道路设施完好率达到98%。进一步加快城市南部地区和站前地区改造，年内改造棚户区100万平方米。开展“城市管理年”活动，对城区环境、市容市貌、交通秩序等进行全方位整治，进一步加大取缔马路市场工作力度，加强小区物业管理，全面提高城市管理水平。做好与群众联系紧密的环境污染治理工作，重点污染企业污染物排放达标率达到100%。采煤沉陷区一期治理工程全部完工，实现安置沉陷区居民18333户的目标，力争二期治理工程开工。

　　实施社会安全工程。全面启动阜新市突发公共事件总体应急预案，创建安全环境。认真落实社会治安综合治理的各项措施，坚决防止恶性案件和事故的发生。深入开展“严打”斗争，依法严厉打击各种刑事犯罪和经济犯罪活动。继续抓好同“法轮功”等邪教组织的斗争。认真做好国家安全工作。要以对国家和人民生命财产高度负责的精神，强化安全生产监督，健全安全生产责任制，强化安全专项整治，切实防止重特大安全事故发生。

　　（九）大力发展科技教育，为经济转型提供科技人才支撑

　　大力增强科技创新能力。继续加强与高等院校、科研院所的科技合作，吸引10家高校院所入驻农业科技成果转化中心。加快发展高新技术产业，实现高新技术产品产值15亿元，增长20%。重点抓好阜新国家农业科技园区农产品加工示范区建设，各类研发中心引进农作物、畜禽新品种（系）100个。在两县五区各建立一个科技培训基地，培训经济转型创业带头人1000名。继续积极推进科技部阜新现代农业科技专项和阜新经济转型科技项目对接洽谈会签约项目建设。

　　积极发展各类教育。充分发挥辽工大在经济转型中的智力优势和人才优势，支持其做大做强。进一步调整地方高等教育布局，集中力量办好阜新高等专科学校。进一步完善中等职业教育布局调整工作，加强农村职教中心建设。巩固普通高中招生规模。新建第三职专教学楼。支持民办教育发展。做好中小学布局调整和人事制度改革工作，积极推进农村九年寄宿制学校建设进程，加强农村初中控辍保学工作。落实市人大常委会《关于阜新市加强教育用地管理议案的决定》，严格管理教育用地。

　　加快人才培育和引进。结合我市经济转型的战略重点，抓紧制定人才培养、引进规划，并将其纳入国民经济和社会发展之中，统筹部署。要着眼于人才总量的增长和人才素质的提高，促进各级各类人才协调发展。继续做好引智工作，全年实施聘请国外专家项目16项，聘请外国专家18人次。

　　（十）推进精神文明和民主法制建设，努力创建和谐稳定的社会环境

　　加强精神文明建设，加快各项社会事业协调发展。以“建设开放阜新，争做文明市民”为主题，深入开展文明城市、文明行业、文明单位创建活动，全面提高市民的整体素质。建设2个省级乡镇文化中心，建设10个农村标准文化室和文化广场。做好新建市图书馆准备工作和影剧院改造规划。广泛开展以篮球项目为重点的全民健身活动，建设篮球学校运动员公寓，抓好篮球、赛艇、举重三个国家级高水平后备人才基地的建设。继续实施有线电视村村通工程，新增入户6万户。全面启动阜新数字电视工程，推广数字电视付费业务。进一步完善突发公共卫生事件应急体系，防保站标准化建设达到100%。易地建设市传染病医院。推进农村新型合作医疗。以创省级卫生城为目标，广泛开展城乡爱国卫生运动。进一步稳定低生育水平，完善农村部分计划生育家庭奖励扶助制度。继续抓好全民国防教育，做好国防动员和民兵预备役、人防、民防工作。深入开展双拥共建活动，搞好优抚安置工作，巩固和发展军政军民团结。做好全国第一次经济普查工作。两县五区全部建立现行文件服务中心。关心和支持老龄、残疾人事业，做好统计、修志、防震减灾等工作。

　　推进民主法制进程，切实维护社会稳定。认真落实市委人大工作会议和政协工作会议精神，自觉接受人民代表大会及其常委会的依法监督和人民政协的民主监督，认真办理人大代表议案、建议和政协提案。加强与民主党派、工商联、无党派人士和人民团体的联系，重视并听取其意见和建议。支持群团组织工作，充分发挥工会、共青团、妇联等群团组织的积极作用。开展以宪法为核心的全民法律知识的普及教育活动，全力做好“四五”普法总结验收工作。继续整顿和规范市场经济秩序，依法严厉打击制售假冒伪劣商品、偷税抗税等违法犯罪行为。开展处理信访突出问题及群体性事件工作专项整治，着重解决工资、社保、农村土地承包征用和违规发包、动回迁等信访热点问题。认真贯彻《民族区域自治法》，全面落实党的民族、宗教政策，增进民族团结，积极支持和帮助少数民族地区经济和社会事业加快发展。继续做好外事、侨务和对台等工作。

　　三、加强政府自身建设

　　新的形势和任务，要求各级政府按照建设开拓创新、公开透明、依法行政和高效廉洁政府的目标，结合实际，切实开展好保持共产党员先进性教育活动，加强行政能力建设，加快推进职能转变和管理创新，切实提高行政效能和服务质量，大力优化发展环境，更好地肩负起阜新转型复兴的历史使命。

　　第一，进一步解放思想，始终保持奋发向上的精神状态。在新一轮思想大解放中，各级政府及其工作人员要坚决克服作风上的守旧松懈，工作上的盲目自满，进一步增强机遇意识、争先意识和创新意识。要从领导干部做起，从具体事情抓起，使各级政府机关成为带头新一轮解放思想、全力支持转型发展的服务型机关。要形成勤于学习、善于思考、肯于琢磨和一抓到底的风气，把主要精力真正放在转型发展上。要抓住新一轮转型发展机遇，着力在发展战略、项目建设、用好用足政策的谋划上下功夫，集中力量编制好“十一五”规划。

　　第二，切实转变职能，把经济发展软环境建设提高到新的水平。加快推进行政管理体制改革，真正把政府经济管理职能转变到主要为市场主体服务和创造良好发展环境上来。要加强和改善对现有企业的服务，规范各类检查和收费行为；进一步降低准入门槛，规范对新办企业的服务行为，所有涉及行政审批项目必须应进全进中心受理，最大限度地减少审批事项和审批环节；成立企业投诉中心，专门受理各类企业对政府部门及授权事业单位服务质量的投诉和举报；对全市行政机关和事业单位全面开展以办事效率、服务质量和工作纪律为主要内容的行政效能监察；开办“走进直播间”栏目，由有关部门主要领导现场解答群众反映强烈的软环境建设问题；健全政务公开制度，政府部门所有应当公开的政策规章、办事程序、审批事项、招标投标等都要及时向社会公开。

　　第三，加强执法队伍建设，提高依法行政的能力和水平。深入贯彻执行《全面推进依法行政实施纲要》，强化行政执法人员的培训考核，不断增强依法行政观念，严格按照法定权限和程序行使权力、履行职责。继续推行行政执法责任制和执法过错追究制，加强行政执法监察，认真解决有法不依、执法不公和多头执法、野蛮执法等群众反映强烈的问题。加强行政执法队伍建设，严肃查处行政“乱作为”和“不作为”现象，坚决清除不合格执法人员。进一步做好行政复议应诉工作，切实保护广大公民、法人和其他组织的合法权益。要不折不扣地贯彻上级作出的决定，做到依法有作为，确保政令畅通。

　　第四，发扬艰苦奋斗作风，建设一支经得起各种考验的诚信、守法、廉洁的公务员队伍。要树立诚信政府形象，对决定的大事、承诺的事情，要说了算、定了干，做到言必行、行必果，以良好的政风取信于民。要重视廉政建设，加强公务员道德、法纪、廉政教育，健全监督约束机制，努力从源头上预防和治理腐败。加强对权力运用各个环节的监督和制约，强化经济转型项目、政府性投入的基本建设工程及事关群众利益的各种专项资金审计监督，在土地出让、工程招标、产权交易、政府采购、资金拨付等方面严格按制度办事，提高领导干部自我约束能力和依法行政能力。深入开展专项治理，坚决纠正部门和行业不正之风。进一步加强市民投诉工作，继续办好“行风热线”栏目。

　　各位代表！阜新的经济转型，为党中央、国务院高度重视，为省委、省政府特殊支持，为全市人民特别关注。对此，我们深感责任重大，使命光荣。让我们紧密团结在以胡锦涛同志为总书记的党中央周围，认真实践“三个代表”重要思想，在中共阜新市委的领导下，紧紧依靠全市人民，凝聚各方力量，开拓进取，奋发图强，在全面推进阜新转型复兴的伟大征程上迈出新的步伐，取得新的更大胜利！
